# Calling non-governmental organisations to strengthen primary health care: Lessons following Alma-Ata

**DOI:** 10.4102/phcfm.v11i1.1945

**Published:** 2019-04-30

**Authors:** Megan Landes, Colin Pfaff, Meseret Zerihun, Dawit Wondimagegn, Sumeet Sodhi, Katherine Rouleau, Michael R. Kidd

**Affiliations:** 1 Dignitas International, Zomba, Malawi; 2 Department of Family and Community Medicine, University of Toronto, Toronto, Canada; 3 Department of Family Medicine, College of Medicine, University of Malawi, Blantyre, Malawi; 4 Department of Family Medicine, Addis Ababa University, Addis Ababa, Ethiopia; 5 College of Health Sciences, Addis Ababa University, Addis Ababa, Ethiopia; 6 Southgate Institute for Health, Society and Equity, Flinders University, Adelaide, Australia; 7 Murdoch Children’s Research Institute, Melbourne, Australia

**Keywords:** global health, family medicine, primary care, primary health care, non-governmental organisations, Alma-Ata

## Abstract

**Background:**

The Alma-Ata Declaration’s commitment to primary health care (PHC) reaches its 40th anniversary in 2018. Over the last 40 years, the number of non-governmental organisations (NGOs) working in low-income countries (LICs) has rapidly multiplied, and over time, NGOs have both positively and negatively impacted equity, effectiveness, appropriateness and efficiency of PHC systems in LICs.

**Aim:**

The authors aim to demonstrate that at the 40th anniversary of the Alma-Ata Declaration’s commitment to PHC, NGOs are particularly poised to strengthen PHC in LICs.

**Methods:**

In this letter, the authors reflect on how NGOs have both positively and negatively impacted equity, effectiveness, appropriateness and efficiency of PHC systems based on their experience working with NGOs in LICs.

**Results:**

NGOs are poised to strengthen PHC in LICs in four distinct ways: assisting with local human resources development, strengthening local information systems, enabling community-based health services and testing innovative service delivery projects.

**Conclusions:**

The authors call for NGOs to commit their expertise and resources to long-term strengthening of PHC in LICs and to critically examine the factors that prevent or assist them in this goal. As the principles of Alma-Ata are renewed, NGOs should be responsibly engaged in strengthening the declaration’s goal of ‘health for all’.

## Letter

At the 40th anniversary of the Alma-Ata Declaration’s commitment to primary health care (PHC) as a path to ‘health for all’, one opportunity that remains to be fully realised is leveraging the efforts of non-governmental organisations (NGOs) in strengthening PHC. Over the last 40 years the number of NGOs working in low-income countries (LICs) has rapidly multiplied, driven by growth in international large-scale funding mechanisms and campaigns to tackle disease-specific epidemics like HIV/AIDS.^1^ This letter reflects on how NGOs have both positively and negatively impacted equity, effectiveness, appropriateness and efficiency of PHC systems from our experience, and it amplifies a call for NGOs to commit their expertise and resources to strengthening PHC in support of ‘health for all’.

Firstly, many LICs face a crisis of inadequate PHC human resources, leading to gross inequities in access to care. While NGOs frequently build capacity of health care workers (HCWs) in LICs, unintended negative consequences of NGOs include increasing internal ‘brain drain’ by drawing skilled HCWs from the public to the better-paid NGO sector, and temporarily taking HCWs from direct service delivery to underserved populations to attend financially incentivised short course training.

However, NGOs can also enhance health equity in PHC services through human resources development. In Ethiopia, to mitigate brain drain, NGOs have joined governments and universities to co-deliver locally designed PHC training programmes that create local and sustainable HCW cohorts with improved retention rates.^2^ To address equity in HCW distribution, NGOs in Malawi have sponsored PHC training of community-chosen rural community members with return-of-service commitments and have focused on on-the-job training within rural communities to improve skills but maintain appropriate HCW coverage.^3^ Meanwhile, in Ethiopia NGOs have worked with the government to support decentralisation of PHC services.^4^

Secondly, NGOs may enhance effectiveness of PHC systems through measuring programme outcomes and supporting rational planning. While health information systems can be weak in LICs, NGOs often invest heavily in robust data collection systems to meet donor requirements. Several examples in Malawi demonstrate that by achieving reporting requirements through strengthening local information systems, NGOs can avoid creating parallel systems and support local data reliability and use in health services planning.^5^

Thirdly, NGOs can be key enablers of community-based health services, enhancing the opportunity of Alma-Ata’s vision to empower communities to manage their own health. While some NGOs still adopt top–down planning or create community-based programmes parallel to the public system, other NGOs have enhanced the context-appropriateness of PHC by integrating with community leadership and addressing community needs. Well-known examples of community-based PHC in sub-Saharan Africa have evolved, such as in Kenya where there is integration of microfinance groups and care for people with chronic diseases, which improves health outcomes by addressing the social determinants of health.^6^ In Ethiopia, numerous NGOs have supported the long-standing health extension worker programme, bringing PHC to traditionally underserved communities.^7^ Efforts should continue to ensure community-based services effectively elicit community participation.

Finally, some NGOs have enhanced efficiency in PHC systems by enthusiastically testing innovations in service delivery. While some criticise a proliferation of NGO small-scale ‘pilot’ projects that have limited potential for scale, examples exist of new innovations successfully navigating scale-up in collaboration with governments. In Malawi and Ethiopia, examples of service delivery innovations include integrating community action groups with HIV services to provide antiretroviral therapy (ART) delivery in rural communities to improve ART outcomes and task-shifting to lay cadres, such as involving HIV ‘expert patients’ in routine clinic duties such as triage and/or patient education, to improve service delivery capacity and ultimately outcomes.^8,9,10^

In 2018, as we look to renew Alma-Ata, critically examining the effects of NGOs on PHC systems in LICs can ensure we can leverage NGOs’ expertise and resources to support ‘health for all’. This includes understanding in more detail how the structure and processes of NGOs inherently inhibit or enhance NGOs’ ability to engage in long-term, locally driven solutions for building strong PHC systems. We amplify the call for a ‘code of conduct’^1^ for NGOs working in LICs and put forth that it should emphasise best practices for prioritising local PHC system strengthening. Recommitting to the principles of Alma-Ata provides an opportunity to engage governments and the NGO sector to explore how to collaboratively improve health equity through strong PHC systems to support ‘health for all’ amongst the people of the world.

## References

[1] Pfeiffer J, Johnson W, Fort M, et al Strengthening health systems in poor countries: A code of conduct for nongovernmental organizations. Am J Public Health. 2008;98(12):2134–2140. 10.2105/AJPH.2007.125989 18923125 PMC2636539

[2] Alem A, Pain C, Araya M, Hodges B Co-Creating a psychiatric resident program for Ethiopians, in Ethiopia: The Toronto Addis Ababa Psychiatry Project (TAAPP). Acad Psychiatry. 2010;34(6):424–432. 10.1176/appi.ap.34.6.424 21041465

[3] Christian Health Association of Malawi (CHAM) Our impact [homepage on the Internet]. CHAM; 2017 [updated 2017, cited 2018 Apr 27]. Available from: http://www.cham.org.mw/our-impact.html

[4] Wamai RG Reforming health systems: The role of NGOs in decentralization – Lessons from Kenya and Ethiopia. 2008 [cited 2018 Apr 27]. Available from: https://c.ymcdn.com/sites/www.istr.org/resource/resmgr/working_papers_barcelona/wamai.pdf

[5] Deutsche Gesellschaft für Internationale Zusammenarbeit (GIZ) GmbH Health systems strengthening with a focus on reproductive health [homepage on the Internet]. [cited 2018 Apr 27]. Available from: https://www.giz.de/en/worldwide/20127.html

[6] Pastakia SD, Manyara SM, Vedanthan R, et al Impact of bridging income generation with group integrated care (BIGPIC) on hypertension and diabetes in rural western Kenya. J Gen Intern Med. 2017;32(5):540–548. 10.1007/s11606-016-3918-5 27921256 PMC5400758

[7] Koblinsky M, Tain F, Gaym A, Karim A, Carnell M, Tesfaye S Responding to the maternal health care challenge: The Ethiopian Health Extension Program. Ethiop J Health Dev. 2010;24(Special Issue 1):105–109.

[8] Pellecchia U, Baert S, Nundwe S, et al ‘We are part of a family’. Benefits and limitations of community ART groups (CAGs) in Thyolo, Malawi: A qualitative study. J Int AIDS Soc. 2017;20(1):21374 10.7448/IAS.20.1.21374 PMC5467612 28406273

[9] Landes M, Thompson C, Mwinjiwa E, et al Task shifting of triage to peer expert informal care providers at a tertiary referral HIV clinic in Malawi: A cross-sectional operational evaluation. BMC Health Serv Res. 2017;17(1):341 10.1186/s12913-017-2291-3 28486980 PMC5423418

[10] Gusdal AK, Obua C, Andualem R, et al Peer counselors’ role in supporting patients’ adherence to ART in Ethiopia and Uganda. AIDS Care. 2011;23(6):657–662. 10.1080/09540121.2010.532531 21347887

